# Cystic hygroma in adults: a single-centre experience and review of the literature

**DOI:** 10.1007/s11845-022-03271-9

**Published:** 2023-01-16

**Authors:** Niall James McInerney, Nick O’Keeffe, Andreea Nae, Juliana Morariu, Con Timon

**Affiliations:** 1 Department of Otolaryngology, Royal Victoria Eye and Ear Hospital, Dublin, Ireland; 2 Department of Otolaryngology, St. James Hospital, Dublin, Ireland

**Keywords:** Cystic hygroma, Lymphangioma

## Abstract

Cystic hygroma (CH) is a benign congenital lymphatic malformation, occurring predominantly in children, typically as an asymptomatic neck mass. Surgical resection or sclerotherapy is the recommended treatment options. A retrospective review of four cases of adult-onset CH was performed over 2 years by a single surgeon across two institutions. Four patients (two females, median age 31.5 years) who presented with supraclavicular neck masses (range 5–17 cm) are discussed. Ultrasound and MRI demonstrated supraclavicular masses, suggestive of CH. All patients underwent surgical resection. Post-operative courses were uncomplicated, with a mean length of stay of 4 days. All histological samples returned as CH. As of yet, there are no guidelines on the management of CH. Individualised care tailored to each patient, following careful discussion is the most prudent approach. This study demonstrates that surgical resection is a safe and effective treatment for adults in this rarely encountered clinical entity.

## Introduction

Cystic hygroma is a benign congenital abnormality, occurring predominantly in newborns [1]. Increased nuchal translucency on fetal ultrasound is typically the first indication of its presence. In newborns, debate persists about the optimal management strategy with a paucity of evidence available [2]. There is sparse guidance on its management in adults.

Adults can present in a variety of ways, but an asymptomatic neck mass is the most likely clinical manifestation [3]. Although benign, treatment is recommended for cosmesis, to prevent recurrent infections and an increase in size [4].

We present our institutional experience in the management of cystic hygromas in adults. In doing so, this can be used a guide for other institutions when faced with this rare clinical entity.

## Methods

A retrospective review of four cases of adult-onset cystic hygroma was performed over 2 years by a single surgeon across two institutions. Here we present the cases detailing the initial presentation, investigations and peri-operative management of cystic hygromas in the adult population in our institution (Table 1).

**Table 1:** Clinical and pathologic features of the reported cases of cystic hygroma

	**Age/gender**	**Presenting complaint**	**Size (cm)**	**Site**	**Imaging**	**Radiological features**	**Macroscopic appearance**	**Length of stay (days)**	**Histology**
**Patient 1**	23 M	Neck mass	17	Right supraclavicular	US, MRI	Multiloculated predominantly cystic mass	Multiloculated cyst filled with yellow/clear fluid	4	Cystic hygroma
**Patient 2**	27 M	Neck mass	12	Right supraclavicular	US, MRI	Well-circumscribed homogenous cyst	Soft cystic lesion filled with yellow fluid	4	Cystic hygroma
**Patient 3**	75 F	Neck mass	10	Left supraclavicular	CT, MRI	Homogenous cystic lesion	Thin-walled cyst filled with cloudy yellow fluid	5	Cystic hygroma
**Patient 4**	36 F	Neck mass	4	Left supraclavicular	US, CT, MRI	Multi-loculated, cystic mass		3	Cystic hygroma

## Case presentations

### Case 1

**Fig. 1:**
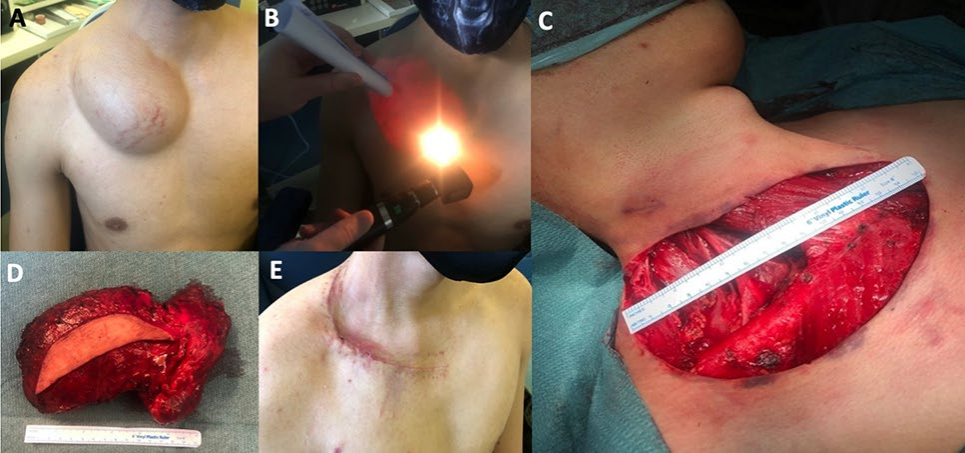
**A** Pre-operative assessment with large right supra-clavicular mass seen. **B** Demonstration of transillumination. **C** Right transverse cervical incision, measuring 15 cm. **D** Gross specimen with cuff of skin attached. **E** Wound review three weeks post-operatively

A 23-year-old male, who was otherwise healthy, was referred to our outpatient department following a 2-year history of a progressively enlarging right supraclavicular swelling. His main concern was cosmesis, and he did not experience symptoms related to airway or oesophageal obstruction. On examination, a 17 cm soft, fluctuant, non-tender, trans-illuminating mass was evident in the supraclavicular region (Fig. 1).

An ultrasound was performed which demonstrated a 10 cm multi-loculated cystic abnormality within the right supraclavicular fossa. Internal septations were seen but it was otherwise anechoic. These radiological appearances were most suggestive of a cystic hygroma.

Further investigation with MRI confirmed a lymphovascular malformation, most consistent with a cystic hygroma.

Following counselling on the potential treatment options, the decision was made to proceed to surgery.

At operation, a transverse incision was made and the strap muscles were divided. The sternocleidomastoid, internal jugular vein, internal carotid artery and vagus nerve were all identified and preserved. The accessory nerve, brachial plexus, and phrenic nerves were preserved. Dissection was performed in a similar fashion to a selective neck dissection, ensuring lymphatics are spared, and that the mass was carefully dissected free ensuring there was no capsular rupture. A drain was placed, and the wound was closed in the standard fashion. The post-operative course was uncomplicated with the patient remaining an inpatient for 4 days.

On examination in the outpatient department 3 weeks postoperatively, the wound had healed satisfactorily. Histology returned as multiloculated mass, with a smooth outer surface and a central cystic scar with yellow clear fluid, most consistent with a large cystic hygroma.

### Case 2

A 27-year-old male was referred to our outpatient department with a right neck swelling which was noticed 6 months previously and had progressively increased in size. He had no past medical history. On examination, a right-sided 12-cm supraclavicular mass, which was soft, fluctuant, and non-tender was evident.

**Fig. 2:**
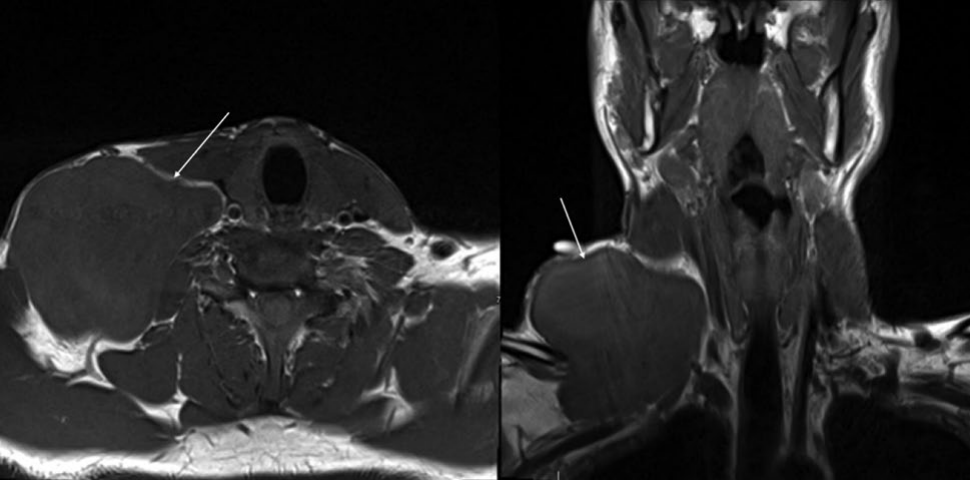
MRI axial and coronal slices demonstrating a large well-circumscribed right cystic mass, postero-lateral to the sternocleidomastoid, and in close proximity to the carotid trunk

An ultrasound demonstrated an 8.6 × 8.3 × 4.7 cm cystic avascular structure with no internal septations. For pre-operative planning, an MRI was performed which showed a well-circumscribed homogenous cyst arising from the right infraclavicular region extending to the supraclavicular region and lower right neck subcutaneous tissues, which was most consistent with a cystic hygroma (Fig. 2).

**Fig. 3:**
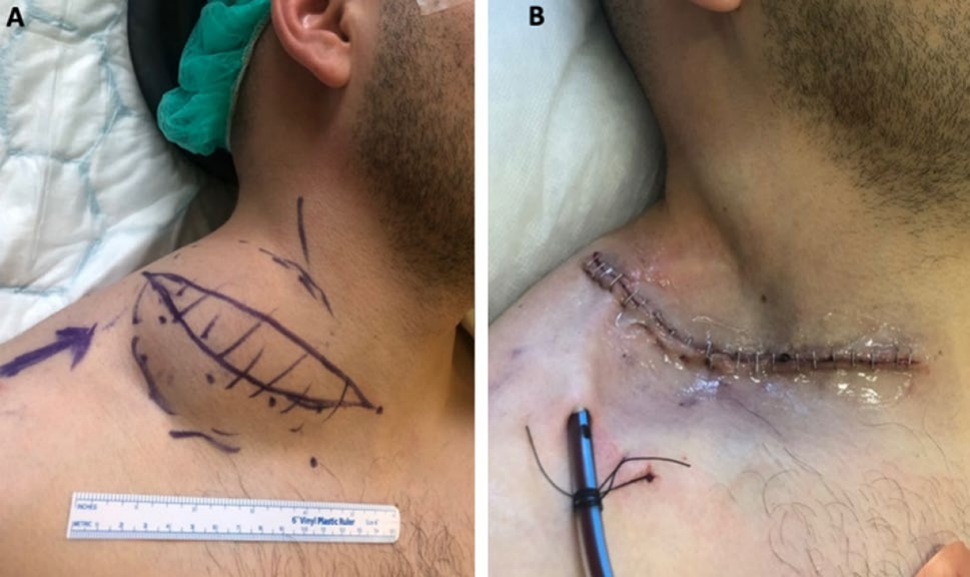
**A** Marking of planned elliptical transverse cervical incision, with the borders of the mass marked. **B** Operative closure with drain in situ

Following this, he proceeded to surgery (Fig. 3). A transverse incision was made and a subplatysmal flap was raised. A cystic mass was seen and dissected free from the sternocleidomastoid and accessory nerve, preserving both structures. The brachial plexus and phrenic nerve were identified and preserved. Again, dissection was performed in a similar fashion to a selective neck dissection and the mass was removed. A closed-suction drain was placed and the wound closed in the standard fashion.

He had an uneventful post-operative course and was discharged on post-operative day four. On review in outpatients 3 weeks post-operatively his wound had healed satisfactorily and histology was consistent with a cystic hygroma.

### Case 3

A 75-year-old female was referred to our institution with a new-onset left-sided neck swelling. Apart from hypertension, she had no other co-morbidities. On examination, a 10 cm soft, transilluminating neck mass was seen.

**Fig. 4:**
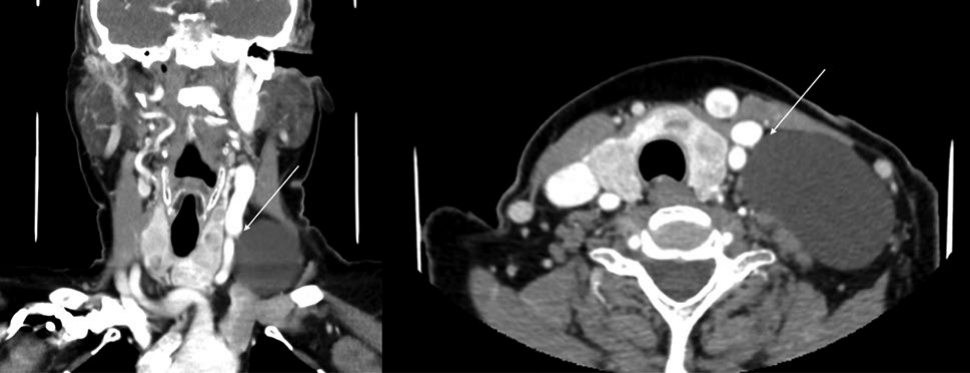
Coronal and axial contrast CT images, demonstrating a homogenous cystic lesion in the left supraclavicular region, in close relationship to the head and neck vasculature

A CT was performed in the referring institution which demonstrated a left supraclavicular homogenous cystic lesion (Fig. 4). An MRI further characterised this as a large septated cystic abnormality.

**Fig. 5:**
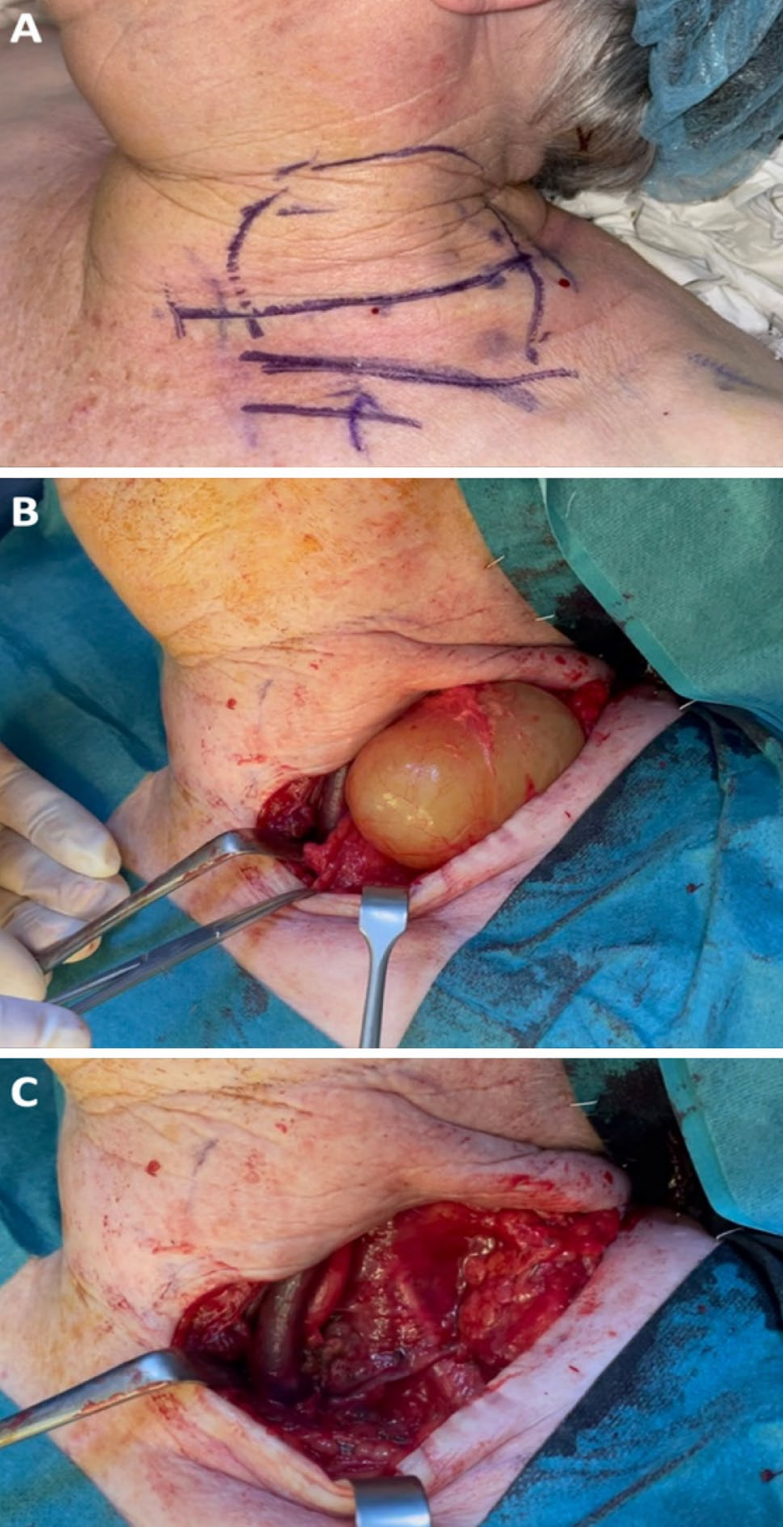
**A** Marking of planned transverse cervical incision, with the borders of the mass marked, and clavicle marker inferiorly. **B** Intra-operative visualisation of large cystic mass dissected free from surrounding structures. **C** Post-removal of the cystic mass

At operation, a transverse incision was made and subplatysmal flaps were elevated. The brachial plexus and left accessory and phrenic nerves were identified and preserved. The cystic mass was dissected free of the carotid sheath and removed intact, without rupture (Fig. 5). A closed suction drain was placed and the wound closed.

She was discharged on post-operative day four and histology returned as a cystic hygroma.

### Case 4

A 36-year-old female was initially referred to our outpatient department for evaluation of a new-onset left neck mass. She was asymptomatic from this and had no comorbidities. An ultrasound was originally performed in the community which demonstrated a 4 cm cystic mass in her left neck, at level 5b.

A CT neck with contrast was performed which demonstrated a multi-loculated, cystic structure posterior to the left external jugular vein. Differentials at this time included a third brachial cleft cyst, cervical dermoid cyst or a cystic hygroma.

In light of this, an ultrasound-guided fine needle aspiration was performed, with serous fluid withdrawn, with cytology suggesting a cystic hygroma. An MRI was undertaken 6 months post-initial presentation. This again demonstrated a 4 × 2 × 3 cm lobulated left supraclavicular lesion in left level 5b, which on MRI most likely represents a lymphatic malformation.

The patient proceeded to surgery. A transverse incision was made and subplatysmal flaps were raised. The brachial plexus, accessory and phrenic nerves were identified and preserved. A cystic mass was clearly visualised and carefully dissected away from surrounding tissue. Haemostasis was ensured and a drain was placed.

She had an uncomplicated post-operative admission and was discharged home on post-operative day three. Histology confirmed a cystic hygroma. On 6-month follow up, she had no evidence of recurrence.

## Discussion

Cystic hygroma is a benign congenital lymphatic malformation, most frequently occurring in the head and neck region. Typically occurring in the neonatal period, but there have been cases of cystic hygromas developing later in life [5, 6]. In infants, it is thought that cystic hygromas develop from the sequestration of lymphatic tissue during the formation of lymphatic-venous sacs, with a failure of the development of the normal lymphatic system [7]. However, debate remains about the aetiology of cystic hygromas in adults, with trauma or infection precipitating delayed proliferation of quiescent lymphatic tissue as the most common hypothesis [8]. Although benign, they can lead to cosmetic deformity and significant morbidity, including pain, infection, feeding difficulty and most notably airway obstruction. In the adult population, resection is important to avoid the misdiagnosis of a cystic metastasis from a well-differentiated papillary thyroid cancer. Still, the most common presentation is an asymptomatic neck mass [9]. Outside of the paediatric population, there is no age predilection for cystic hygromas [3].

Ultrasound is used as first-line imaging, particularly to differentiate between a cystic mass and solid tumours. CT is used to delineate relevant anatomic structures but if available MRI provides greater soft tissue and anatomic differentiation [3, 10]. Pre-operative staging can be performed in children based on anatomic location [11]. A greater pre-operative stage has been correlated to a worse prognosis and increased morbidity [1]. No adults were included in the study [11].

Initially, surgical resection was the mainstay of treatment, but debate arose following the advent of sclerotherapy [2]. Sclerotherapy involves injecting a sclerosant into the cyst which causes inflammation, thrombosis and ablation of the cyst [12]. A variety of agents have been used including bleomycin, doxycycline, ethanol and OK-432. Prior to injection, it is important to be aware of the risk of anaphylaxis. Sclerotherapy often requires multiple procedures with a delayed response time [13]. If unsuccessful and surgery is required, scarring and fibrosis lead to a more challenging surgical resection. By the same logic, aspiration of the cyst should be avoided. There are no randomised control trials comparing surgery to sclerotherapy; however, on retrospective review in children, surgery has been shown to be a more effective management strategy [14]. However, due to its location near significant neurovascular structures, surgery can be challenging, and if incompletely resected or ruptured, the residual cyst can precipitate recurrence [15].

While there has been no evidence of recurrence in our patient cohort, there have been reports of a 10–27% recurrence in completely excised lesions, with a 50–100% risk of recurrence in incompletely excised lesions [16, 17]. It is imperative to counsel patients on the potential risk of recurrence and damage to neurological structures prior to surgical intervention. It is our view that dissection should be performed in a similar fashion to a selective neck dissection, ensuring there is no capsular rupture.

As of yet, there are no guidelines on the management of cystic hygromas [2]. Individualised care tailored to each patient, following careful discussion is the most prudent approach. All patients in our cohort presented with asymptomatic neck masses, with no evidence of obstructive symptoms. In each case, surgical resection was performed with cystic masses removed completely without rupture. No peri-operative morbidity was recorded in this study. At 6-month follow-up, no evidence of recurrence was evident in all cases.

This study demonstrates the safe effective use of surgical resection to treat cystic hygroma in the adult population. Surgery provides a safe and effective treatment option, with a low risk of peri-operative morbidity.

## Data Availability

All data generated or analysed during this study are included in this published article (and its supplementary information files)
